# Iodine status five years after the adjustment of universal salt iodization: a cross-sectional study in Fujian Province, China

**DOI:** 10.1186/s12937-021-00676-7

**Published:** 2021-02-23

**Authors:** Yixuan Lin, Diqun Chen, Jiani Wu, Zhihui Chen

**Affiliations:** 1 Fujian Center for Disease Control and Prevention, Department of Endemic Diseases, Fujian, No. 76 Jintai Road, Fujian 350001 Fuzhou, People’s Republic of China; 2 Department of Global Public Health, Karolinska Institutet, Tomtebodavägen 18, 17165 Solna, Sweden; 3 School of Public Health, Fujian Medical University, University of New Area, No.1 Xueyuan Road, Fujian 350108 Fuzhou, People’s Republic of China

**Keywords:** Adjustment, Iodized salt, Urine iodine concentration, Iodine status

## Abstract

**Background:**

Universal salt iodization program was introduced to China to eliminate iodine deficiency disorders in 1995. In 2012, Fujian Province decreased the concentration of iodized table salt according to the national unified requirement. This study aimed to assess the effect on iodine status after the adjustment, providing evidence for further adjustment in Fujian Province.

**Methods:**

Sampling units were selected by multistage cluster sampling method. In each sampling unit, table salt was collected from 30 households. A total of 2,471 people in 2009 and 4,806 people in 2017 provided urine samples and were included in this cross-sectional analysis. Median iodized salt concentration and median urine iodine concentration were present by median and interquartile range.

**Results:**

Median iodized salt decreased from 29.8 mg/kg in 2009 to 23.9 mg/kg in 2017. The median urinary iodine concentrations for school-age children in 2017 in coastal urban area, non-coastal urban area, coastal rural area and non-coastal rural area were 163.6µg/L (interquartile range = 100.1–252.0µg/L), 198.9µg/L (interquartile range = 128.0-294.0µg/L), 181.8µg/L (interquartile range = 114.1–257.0µg/L) and 218.2µg/L (interquartile range = 148.1-306.5µg/L), respectively. The median urinary iodine concentrations for adults in 2017 in these areas were 151.1µg/L (interquartile range = 98.3-231.7µg/L), 168.7µg/L (interquartile range = 109.6–242.0µg/L), 167.7µg/L (interquartile range = 105.7-245.7µg/L) and 182.7µg/L (interquartile range = 117.1-258.9µg/L). The median urinary iodine concentrations for pregnant women in 2017 in these areas were 157.7µg/L (interquartile range = 106.9-223.8µg/L), 141.5µg/L (interquartile range = 97.7-207.6µg/L), 127.3µg/L (interquartile range = 90.0-184.5µg/L) and 144.8µg/L (interquartile range = 99.9-184.5µg/L). The median urinary iodine concentrations for lactating women in 2017 in these areas were 122.7µg/L (interquartile range = 84.1–172.0µg/L), 123.7µg/L (interquartile range = 70.7-184.7µg/L), 105.8µg/L (interquartile range = 67.1-152.3µg/L) and 110.2µg/L (interquartile range = 74.1-170.3µg/L).

**Conclusions:**

The overall urinary iodine concentrations among school-age children, adults and lactating women dramatically decreased after implementing the new standard. Almost all of them were iodine adequate, suggesting we reached the expected aim of iodized salt adjustment. However, pregnant women were iodine insufficient after adjustment. Therefore, we should continue the surveillance of iodine status of populations and focus on the additional iodine supplement strategies for pregnant women.

## Introduction

Iodine is an essential element for thyroid hormones production, playing an important role in our early life, influencing human’s neurodevelopment, growth and cellular metabolism [1, 2]. Lack of iodine can cause iodine deficiency disorders (IDD), including abortion, stillbirth among fetuses, endemic cretinism among neonate, delayed physical development among children and adolescents and endemic goiter among all age groups. Excess iodine can also cause side effects, such as hypothyroidism and autoimmune thyroid [3, 4].

Iodine deficiency is a public health issue in the world [5], and it was also a public health issue in China before 1995 [6], becoming an obstacle on the way of Chinese economic development and national quality improvement. Some developed countries choose bread and milk as carriers while China chooses salt as the most cost-effective and simplest carrier [7–10]. In 1995 China launched the Universal salt iodization program aiming for improving health for citizens particularly among children and women. After the implementation of this program, people’s iodine intake mainly comes from iodized salt. By 2000, the Chinese Ministry of Health announced that China had reached the goal of eliminating IDD. However, the national iodine surveillance pointed out that the median urinary iodine concentration (mUIC) in some provinces was above 300µg/L if the iodized salt concentration was between 40 and 60 mg/kg. Then the government adjusted the standard of the concentration from 40 to 60 mg/kg to 20-50 mg/kg [6]. However, a couple of provinces were still maintained a relatively high mUIC because of the high-salt diet, and there were some possibilities for a small its reduction [11]. Given that salt consumption differs in different geographical areas, each province has been authorized to set its concentration criterion based on their geographical factors and population iodine status since March 2012. Fujian set its criterion between 18 and 33 mg/kg.

This study aimed to assess the iodine status in Fujian Province 5 years after the adjustment of universal salt iodization, thereby investigating the appropriateness of iodine status between populations, the differences of iodine status before and after the adjustment, evaluating the rationality of the adjustment and trying to give some suggestions for the iodized salt concentration adjustment.

## Methods

### Study design and participants

This cross-sectional study employed the multistage cluster sampling method in 2009 and 2017. There are 9 administrative regions in Fujian Province. An urban area and a rural area were randomly selected from each administrative region. One district was randomly selected from an urban or rural area; one town was randomly selected from the selected district; one village was randomly selected from the selected town. The 18 sampling units were classified as coastal or non-coastal units based on their geographical location. There were 4 units classified into coastal urban area, 5 units classified into non-coastal urban area, 6 units classified into coastal rural area and 3 units classified into non-coastal rural area.

The participants from the villages were recruited, and the inclusion criteria were who were: (1) healthy; (2) local residents. The exclusion criteria were who: (1) lived in this area less than 5 years; (2) were receiving or treated with amiodarone; (3) with abnormal renal function; (4) used coronary angiography or endoscopic retrograde cholangiopancreatography within 6 months. In 2009 and 2017, thirty households were randomly selected from each village, a salt sample with at least 30 g was collected from a household, and the samples were protected from the light until the detection. In 2009, 20 adults aged 18 to 45 were randomly selected from each sampling unit, half male and half female. 30 pregnant women, 30 lactating women and 50 school-age children (half male and half female) from one elementary school aged 8 to 10 were randomly selected from the sampling unit as well. In 2017, 60 adults aged 20 to 50 were selected from each sampling unit, half male and half female. 50 pregnant women, 50 lactating women and 100 school-age children half male and half female from one elementary school aged 8 to 10 were randomly selected from the sampling unit as well. 5 ml spot urine samples were collected from the participants, and the samples were stored at 4℃ in transit. The urinary samples were processed and their urinary iodine concentrations (UIC) were measured by acid digestion method (WS/T107- 2006) [12], and the salt samples were measured by colorimetric titration method (GB/T13025.7-2012) [13] in the laboratory of Fujian Centre for Disease Control and Prevention (CDC). Low, medium and high concentration of reference materials were used in the assay which was provided by China National Iodine Deficiency Disorders Reference Laboratory.

### Assessments

Since Fujian’s regulated concentration of iodized salt in is 25 mg/kg after the adjustment, the iodine concentration of qualified table salt should fall in the range of 18–33 mg/kg while the national standard before 2012 was 35 mg/kg with the range of 20–50 mg/kg. To assess iodine status in populations, World Health Organization (WHO) recommends median UIC (mUIC) as an index. There are 4 iodine statuses for school-age children and adults: insufficient (mUIC < 100µg/L), adequate (100µg/L ≤ mUIC ≤ 199µg/L), above requirements (200 ≤ mUIC ≤ 299µg/L), and excessive (mUIC ≥ 300µg/L). For pregnant women, they have other 4 cut-off points: insufficient (mUIC < 150µg/L), adequate (150µg/L ≤ mUIC ≤ 249µg/L), above requirements (mUIC ≤ 250µg/L ≤ 499µg/L), and excessive (mUIC ≥ 500µg/L). For lactating women, mUIC < 100µg/L was defined as insufficient, and mUIC ≥ 100µg/L was defined as adequate [14].

### Quality control

All the investigators received unified training before the investigation, and they should clearly understand the items of the questionnaire and fill in the questionnaires carefully. Missing items should be avoided. Clean polyethylene containers with caps were used. The correlation coefficient of the standard curve of urine iodine concentration was required to be above 0.999. Standard substances were used before, during and after the tests.

### Statistical analysis

All the data was entered into the database by EPI DATA 3.5.1 software, and the analysis was conducted by SPSS software (SPSS 20.0; IBM Corp, Armonk, NY, USA). The sample size of each area was determined by the variation of UIC; in 2009 the variation of mUIC was set to 20 %, the sample size should be greater than 31; in 2017 the variation of mUIC was set to 10 %, the sample size should be greater than 122 [15]. Since UIC was abnormally distributed, Wilcoxon test was conducted to compare the UIC in each population and area before and after the adjustment, and the UIC was reported as median (M) and interquartile range (IQR, Q_1_ ~ Q_3_), adults ages were reported as mean ± standard deviation (SD). All the tests were two-sided and *P* < 0.05 was considered statistically significant.

The authors are accountable for all aspects of the work in ensuring that questions related to the accuracy or integrity of any part of the work are appropriately investigated and resolved.

## Results

### Sample size by population and geographical locations

Four thousand eight hundred six people participated in the study in 2017 while 2,471 people participated in the study in 2009 (Table 1).

**Table 1:** Number of samples by population and geographical locations in 2009 and 2017

Location	Schoo-age childrenl (n)		Adults (n)		Pregnant women (n)		Lactating women (n)	
	2009	2017	2009	2017	2009	2017	2009	2017
Coastal urban area	155	418	61	255	90	203	93	200
Non-coastal urban area	212	505	158	310	110	253	98	250
Coastal rural area	400	631	163	363	242	313	254	301
Non-coastal rural area	160	315	63	185	101	150	111	154
Total	927	1869	445	1113	543	919	556	905

### Demographic characteristics of school-age children and adults

Nine hundred twenty-seven school-age children and four hundred forty-five adults participated in this study in 2009 while 1,113 school-age children and 1,869 adults participated in this study (Table 2).

**Table 2:** Demographic characteristics of school-age children and adults in the year of 2009 and 2017

	2009	2017
School-age children		
** Sex**		
Male, n (%)	469 (50.6)	962 (51.5)
Female, n (%)	458 (49.4)	907 (48.5)
** Age (years)**		
8, n (%)	225 (24.3)	611 (32.7)
9, n (%)	378 (40.8)	739 (39.5)
10, n (%)	324 (35.0)	519 (27.8)
**Adults**		
** Sex**		
Male, n (%)	220 (49.4)	561 (50.4)
Female, n (%)	225 (50.6)	552 (49.6)
**Age (years), mean ± SD**	33.6 ± 7.6	35.4 ± 8.5

*SD* standard deviation

### Iodine concentration in salt before and after the adjustment

**Fig. 1:**
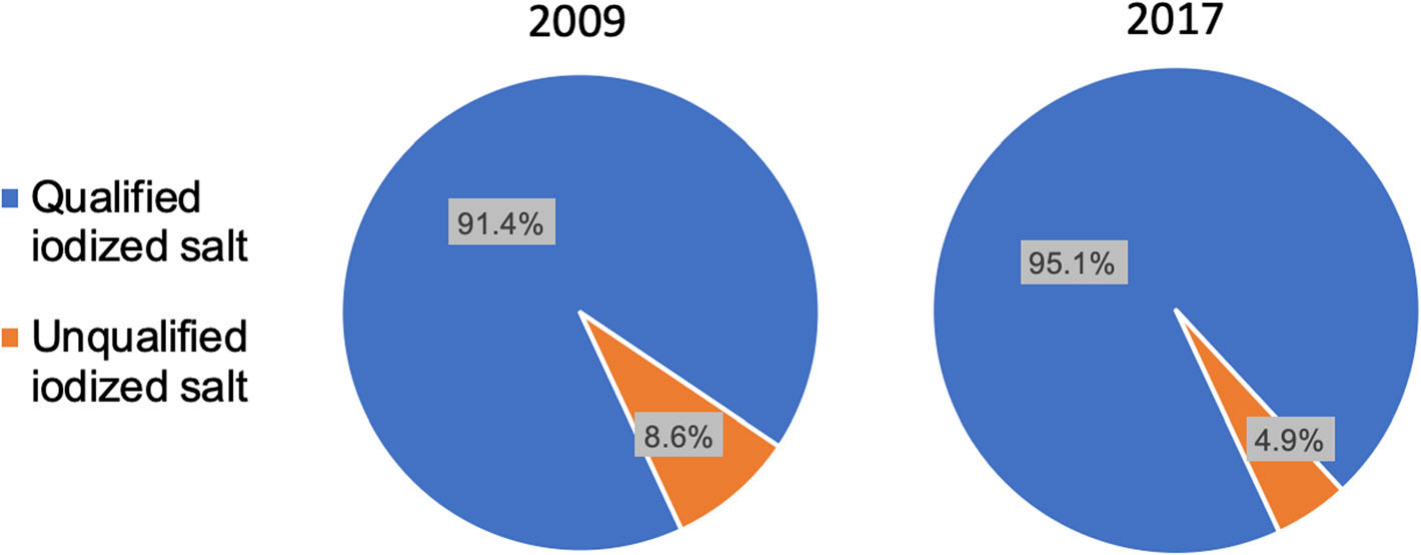
Percentage of qualified and unqualified iodized salt collected from household in 2009 and 2017. The left pie chart is the percentage of qualified and unqualified iodized salt in 2009 (*n*=522), the right pie chart is the percentage of qualified and unqualified iodized salt in 2017 (*n*=566)

The median iodine concentration of table salt was 29.8 mg/kg (IQR = 27.0-31.9 mg/kg) in 2009 and 23.9 mg/kg (IQR = 22.6-25.0 mg/kg) in 2017 (Fig. 1).

### Median urinary iodine concentration distribution after the adjustment

**Fig. 2:**
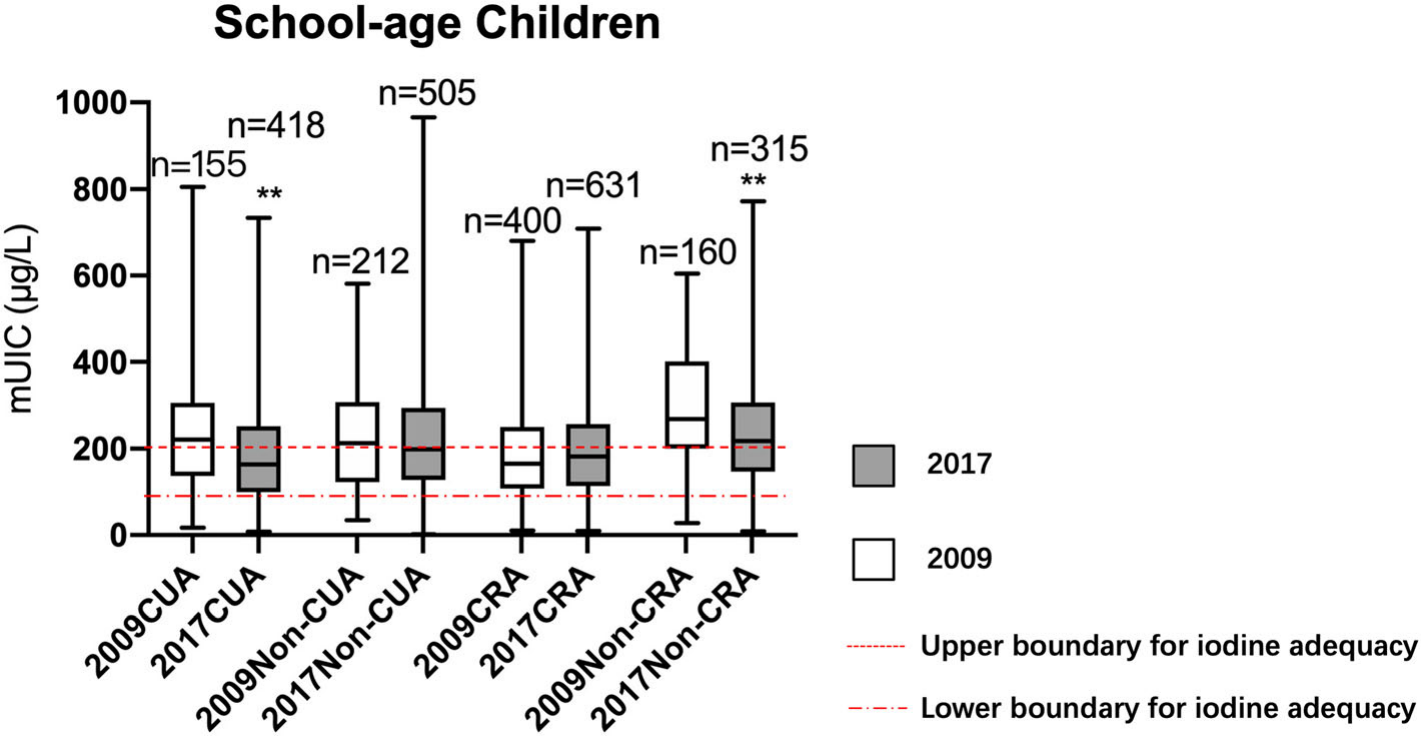
Comparison of urinary iodine concentration by geographical locations in 2009 and 2017 among school-age children. mUIC: urinary iodine concentration; CUA: coastal urban area; Non-CUA: non-coastal urban area; CRA: coastal rural area; Non-CRA: non-coastal rural area. ^**^Urinary iodine concentration (UIC) in 2017 was significantly lower compared to 2009 in each area (*P*<0.05) by Wilcoxon test (*P*<0.001)

In 2017, for overall school-age children, significantly lower mUICs were observed in the coastal urban area (*P* < 0.001) and non-coastal rural area (*P* < 0.001) compared to 2009 (Fig. 2). mUIC of school-age children in the non-coastal rural area was iodine above requirement (218.2µg/L, IQR [interquartile range] = 148.1-306.5µg/L); pregnant women in non-coastal urban area, coastal rural area and non-coastal area were iodine deficiency (141.5µg/L [IQR = 97.7-207.6µg/L], 127.3µg/L [IQR = 90.0-184.5µg/L] and 144.8µg/L [IQR = 99.9-184.5µg/L], respectively). Moreover, the rest of the subjects in each subgroup were iodine sufficient in 2017. The mUIC of school-age children in the non-coastal rural area (218.2 µg/L [IQR = 148.1-306.5µg/L]) was still acceptable although it exceeded the cut-off point of 200µg/L.

**Fig. 3:**
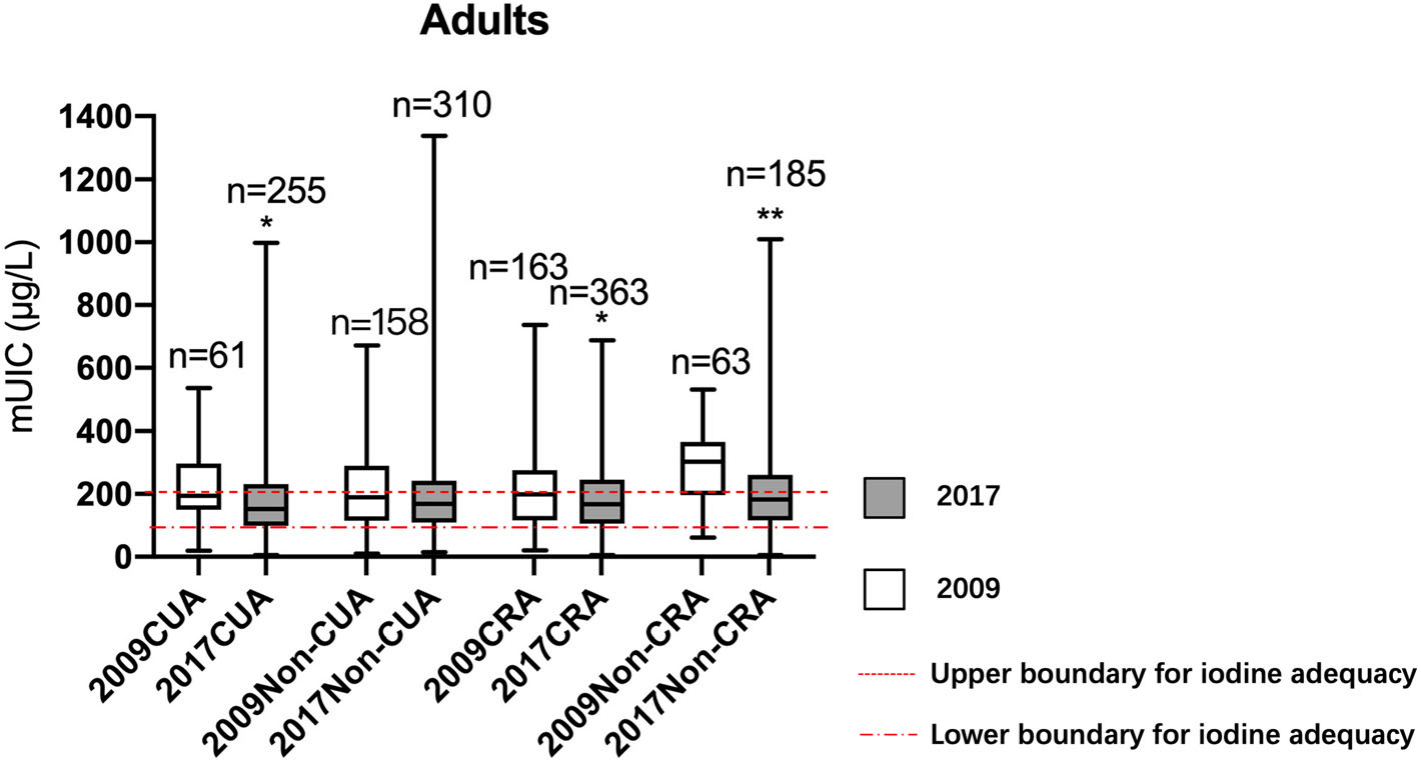
Comparison of urinary iodine concentration by geographical locations in 2009 and 2017 among adults. mUIC: median urinary iodine concentration; CUA: coastal urban area; Non-CUA: non-coastal urban area; CRA: coastal rural area; Non-CRA: non-coastal rural area. ^*^Urinary iodine concentration (UIC) in 2017 was significantly lower compared to 2009 in each area (*P*<0.05) by Wilcoxon test. ^**^Urinary iodine concentration (UIC) in 2017 was significantly lower compared to 2009 in each area (*P*<0.05) by Wilcoxon test (*P*<0.001)

For adults, the overall iodine status was significantly lower in 2017 (166.1µg/L [IQR = 104.9-243.8µg/L]) compared to 2009 (199.9µg/L [IQR = 129.0-304.6µg/L]) (*P* < 0.001) (Fig. 3).

**Fig. 4:**
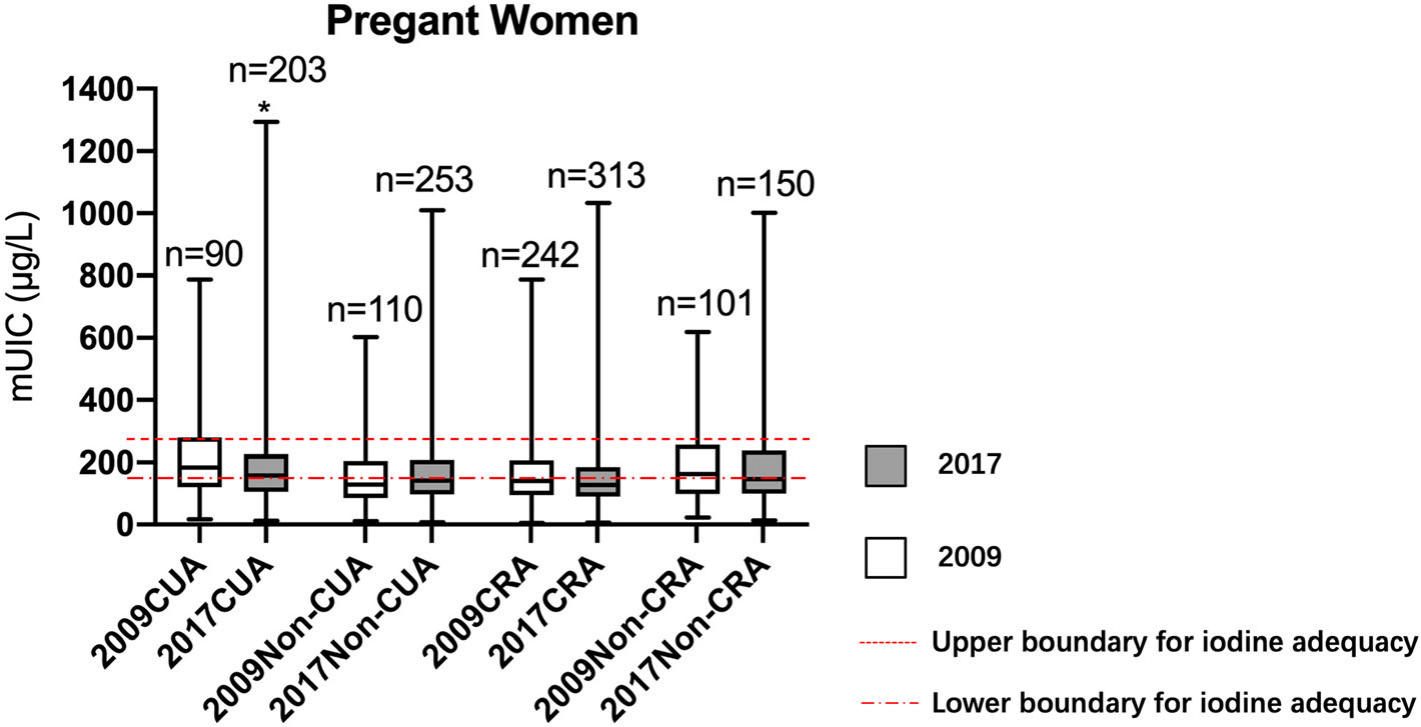
Comparison of urinary iodine concentration by geographical locations in 2009 and 2017 among pregnant women. mUIC: median urinary iodine concentration; CUA: coastal urban area; Non-CUA: non-coastal urban area; CRA: coastal rural area; Non-CRA: non-coastal rural area. ^*^Urinary iodine concentration (UIC) in 2017 was significantly lower compared to 2009 in each area (*P*<0.05) by Wilcoxon test

For overall pregnant women, there is no significant difference between two years (*P* = 0.21), and the mUICs of pregnant women in non-coastal urban area, coastal and non-coastal rural areas were iodine deficiency after adjustment (141.5µg/L [IQR = 97.7-207.6µg/L], 127.3µg/L [IQR = 90.0-184.5µg/L] and 144.8 µg/L [IQR = 99.9-184.5µg/L]) (Fig. 4).

**Fig. 5:**
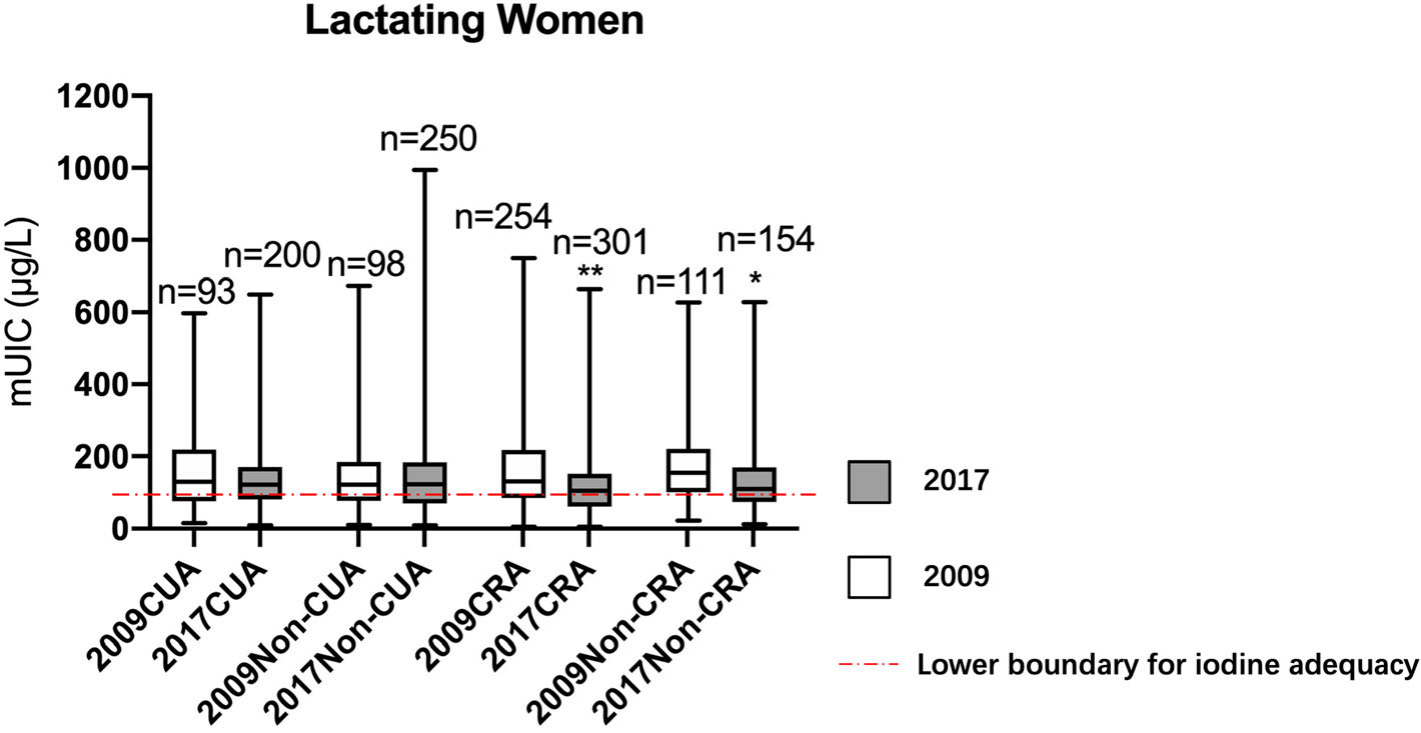
Comparison of urinary iodine concentration by geographical locations in 2009 and 2017 among lactating women. mUIC: median urinary iodine concentration; SAC: school-age children; CUA: coastal urban area; Non-CUA: non-coastal urban area; CRA: coastal rural area; Non-CRA: non-coastal rural area. ^*^Urinary iodine concentration (UIC) in 2017 was significantly lower compared to 2009 in each area (*P*<0.05) by Wilcoxon test. ^**^Urinary iodine concentration (UIC) in 2017 was significantly lower compared to 2009 in each area (*P*<0.05) by Wilcoxon test (*P*<0.001)

For lactating women, the overall iodine status was significantly lower in 2017 (113.8µg/L [IQR = 69.6-171.1µg/L]) after the adjustment (*P* < 0.001), and the iodine status was adequate across all area groups (Fig. 5).

## Discussion

The results from this study showed that the coverage of qualified iodized salt in 2017 remained above 90 %, and the median iodine concentration significantly decreased from 29.8 mg/kg in 2009 to 23.9 mg/kg in 2017. The results also revealed that the mUICs among school-age children and adults were 100–200µg/L in 2017, indicating that the iodine status was adequate with the exception for the above requirements of non-coastal rural school-age children. Although mUICs of non-coastal rural school-age children was still above requirements, iodine concentration experienced a dramatic decrease and finally it was close to the upper boundary of adequacy, suggesting that we reached the expected aim of iodized salt adjustment. However, there was no significant decrease in overall UIC for pregnant women after the adjustment, and only the coastal urban area met the criterion set by WHO among the 4 geographical areas. And mUICs for lactating women remained at an adequate level albeit there was a significant decrease after the adjustment.

The reduced concentration was based on the iodine status among the local people in each province. A total of 13 provinces like Fujian, Zhejiang and Guangxi reduced their concentration of iodized salt from 35 mg/kg to 25 mg/kg. Similarly, the mUIC of school-age children in Zhejiang Province fell to 174.3µg/L [16]; Guangxi Province which reduced its iodine concentration to 25 mg/kg had the similar tendency as well [11]. Yet 11 provinces like Shanghai set their standard of 30 mg/kg [17]. In addition, some provinces set a combined standard - higher iodine concentration for pregnant women and lower iodine concentration for the other population groups [6].

In this investigation, over 90 % coverage of qualified iodized salt after adjustment indicated that our province still satisfied the requirement of eliminating IDD, and the difference before and after the adjustment was statistically significant suggesting that this reduction was in place. Nonetheless, the median iodine concentration of table salt was below the theoretical value, especially in the year of 2009. We supposed that the factories lowered the iodine concentration with the purpose of cost reduction as long as it was still within the interval. Certainly, they could not avoid the loss of iodine in production which seemed to be another reason. We also noticed that the mUICs in 2017 were much higher in school-age children (186.5µg/L) and adults (165.8µg/L) compared to pregnant women (141.4µg/L). The reasons for deficiency among pregnant are: (1) thyroid hormone production increases by 50 % during gestation [18]; (2) iodine is given priority to fetus in order to satisfy the fetus’s nervous system development [19]; (3) the iodine renal clearance during pregnancy increases [19–21]; (4) T_3_ deiodinase activity increases [19]. In addition, vomit during pregnancy could partly cause iodine loss. Also, some pregnant women chose a low-salt diet in the prevention of edema and pregnancy-induced hypertension. Another finding was that in 2017 almost all the mUICs was higher of each population in non-coastal area than coastal area wherever they were rural or urban, and we suppose it may be associated with the salty diet among non-coastal residents.

The results suggest that the adjustment was suitable for school-age children, adults and lactating women. The mUICs in 2017 among school-age children, adults and lactating women significantly declined but still maintained at the adequate level after the adjustment of iodine concentration. The results showed that the mUIC among lactating women was still above the cut-off point of iodine adequate based on criterion by WHO/United Nations International Children’s Emergency Fund (UNICEF) [11], but there was a substantial decline especially for the rural women, the median urinary iodine was only 105.8µg/L for coastal area and 110.2µg/L for non-coastal area, which closed to the cut-off point. Given that iodine for the new-borns is mainly from lactating women and lactating women are of a high risk of iodine deficiency, the United States The Pediatrics Society recommends that lactating women should take iodine supplements at least 150 µg per day [22]. Thus, it is essential to monitor and assess their iodine status or even adopt some strategies target at lactating women.

However, there was no significant difference in mUIC among pregnant women - the mUIC was below 150µg/L both before and after the adjustment, defined as iodine deficient according to criterion recommended by WHO/UNICEF [11]. Hence, we can say that the adjustment had its limited impact on pregnant women. Similarly to Fujian and other province, the pregnant women in some developed countries face iodine deficiency as well, such as Austria, Norway and Sweden [23, 24]. Studies have shown that iodine deficiency during pregnancy can cause adverse effects on the nervous system [25–28] and psychiatric development of the offspring [25, 29]. The earlier the iodine supplementation of pregnant women, the better the mental development of the offspring [30]. As it is difficult for pregnant women to satisfy the iodine requirements by iodized salt only, we suggest iodine supplement tablets (150µg/day) as most European countries [31] and the enriched iodized food intake, such as laver and kelp.

 The primary strength of the study is the large sample size - we recruited 2,471 participants in 2009 and 4,806 participants in 2017. A limitation of this study is that UICs could be influenced by water consumption which can differ per season as people tend to drink more in summer and less in winter, and the current study collected the samples between December to April. Secondly, iodine status was assessed by spot urine sample which could be greatly affected by laver and kelp, followed by shellfish while other seafood does not contain so much iodine; if some participants ate these seafood on the day of sampling, as a consequence, their urine would be detected with high iodine concentration, so spot urine sample cannot fully represent the actual daily iodine consumption.

## Conclusions

Our findings suggest that the iodine status, in general, is adequate among school-age children, adults and lactating women after the adjustment of universal salt iodization program in Fujian Province, indicating the current iodized salt concentration is more appropriate for the populations and we reached the aim of iodized salt adjustment. However, pregnant women were iodine insufficient after adjustment so additional measures should be taken. Iodized salt, as the major approach of iodine supplement in our country, should be continuously promoted in our province even in coastal area and islands.

## Data Availability

The datasets used during the current study are available from the corresponding author on reasonable request.

## Abbreviations

IDD: Iodine deficiency disorders
mUIC: Median urine iodine concentration
UIC: Urine iodine concentration
CDC: Centre for Disease Control
WHO: World Health Organization
IQR: Interquartile range
UNICEF: United Nations International Children’s Emergency Fund
